# Moderate intensity active recovery improves performance in a second wingate test in cyclists

**DOI:** 10.1016/j.heliyon.2023.e18168

**Published:** 2023-07-11

**Authors:** Marco Gervasi, Eneko Fernández-Peña, Antonino Patti, Piero Benelli, Davide Sisti, Johnny Padulo, Daniel Boullosa

**Affiliations:** a Department of Biomolecular Sciences - Division of Exercise and Health Sciences, University of Urbino Carlo Bo, Urbino, Italy; b Department of Physical Education and Sport, University of the Basque Country UPV/EHU, Vitoria-Gasteiz, Spain; c Sport and Exercise Sciences Research Unit, Department of Psychology, Educational Science and Human Movement, University of Palermo, Palermo, Italy; d Department of Biomedical Sciences for Health, Università Degli Studi di Milano, Milan, Italy; e Universidad de León, León, Spain; f College of Healthcare Sciences, James Cook University, Townsville, Australia

**Keywords:** Oxygen consumption, Power output, Lactate clearance, Warm-up, Post-activation performance enhancement

## Abstract

**Background:**

The aim of the present study was to compare the effects of active (AR) vs. passive recovery (PR) between two Wingate Anaerobic Tests (WAnT) on power output, blood lactate (BLa) and oxygen consumption (VO_2_) in a second WAnT.

**Methods:**

Twelve well-trained cyclists underwent three experimental sessions. In the first session, they completed an incremental test for maximum oxygen consumption (V O_2_max) and lactate threshold determination. In the second and third sessions, cyclists completed, in random order, two WAnT tests separated by 30-min recovery intervals, during which they performed an AR at 70% of the V O_2_ at lactate threshold (V O_2_LT) or a PR. The cardiorespiratory, metabolic, and mechanical responses in the two recovery conditions were compared.

**Results:**

No differences were found in the VO_2_-on kinetics between WAnT tests (p > 0.05). As expected, blood lactate kinetics showed a greater clearance (from the 7th to the 31st min, p < 0.001) during AR; however, no differences were found in peak BLa between conditions (p > 0.05). Mean and peak power, and total work were significantly higher in the second WAnT after AR (p < 0.001), while the power decline was also lower in this condition (p < 0.05).

**Conclusion:**

The submaximal active recovery strategy used in the present study can induce an improvement in mechanical power and total work during a second WAnT. This suggests that AR of submaximal intensity can induce a post-activation performance enhancement when used during the recovery phase between maximal anaerobic efforts.

## 1 Introduction

It is widely recognized that the warm-up preceding high-intensity exercise is vital to achieving optimal performance. One of the main outcomes associated with warming up is an increase in body temperature which is accompanied by increases in muscle metabolism and muscle fiber conduction velocity [1,2]. Elevated VO_2_ kinetics [3] and increases in muscle contractile performance following prior contractile activity [4] have also been reported [5]. The use of different warm-up strategies to improve neuromuscular performance has been suggested to enhance muscle power performances through post-activation performance enhancement (PAPE). Another warm-up strategy for high-intensity exercises is the so-called “priming exercise”, which consists of a high-intensity warm-up completed above the lactate threshold. The priming exercise has shown a faster overall VO_2_ kinetics during a second heavy-intensity exercise bout, and several investigations [6,7] have established that prior heavy-intensity exercise may reduce the overall O_2_ deficit, as a result of an acceleration of the primary component and a reduced slow component amplitude of VO_2_ kinetics. As a result, there is a greater O2 availability in the working muscles and thus an increase in energy supply and exercise intensity can be expected [8]. Therefore, warm-up strategies before high-intensity exercises should include exercises, not only with the aim of increasing body temperature, but also aiming to enhance neuromuscular performance and VO_2_ kinetics via PAPE and priming exercises, respectively. However, previous literature has separately analyzed the metabolic and neuromuscular effects of different warm-up strategies on subsequent high-intensity exercises. On the other hand, recovery strategies after high-intensity exercises are well documented in the literature. The two main strategies are active (AR) and passive recovery (PR). Literature suggests that when the exercise is an “all out” effort lasting <15 s, the best recovery strategies should be passive [[9], [10], [11]]. Indeed, it has been also reported that an AR between a series of 4-s maximum sprints has a negative impact on performance, whereas PR helps to maintain performance [9]. In contrast, if the high-intensity exercise lasts between 15 s and 1 min, it has been suggested that AR is more effective in maintaining the performance of subsequent bouts [[12], [13], [14]]. Furthermore, previous studies have also shown a performance enhancement when an AR was performed between two high-intensity activities of a duration ≥30 s [[15], [16], [17]]. These previous studies have focused more on blood lactate responses, the increases in VO_2_peak and the excess of CO_2_ found during the second sprint after an active recovery at 30 and 50% of the VO_2_ at the ventilatory threshold, therefore suggesting that the AR improved the oxidative energy supply during the second sprint [16,17]. However, the AR intensity adopted between the two exhaustive exercises in these previous studies was lower than the optimal since other authors previously suggested that the optimal intensity related to the blood lactate removal rate should be at 80% of LT, while that related to the working capacity should be at 70% of LT [18,19]. However, these previous studies have been conducted with non-cyclists. The simultaneous evaluation of muscle power, lactate and VO_2_ kinetics during supramaximal efforts after passive vs. active recovery modes has not been yet addressed in trained cyclists. This information would be relevant to cyclists competing at the UCI Track Cycling World Championships who spend ∼30–40 min between sprinting heats. Thus, the aim of the current study was to assess the effects of active recovery (70% of VO_2_LT) vs. passive recovery on the mechanical and metabolic variables during the execution of two Wingate anaerobic tests (WAnT) in competitive cyclists. Based on previous literature, our hypothesis was that an AR would enhance the lactate clearance during the recovery phase while increasing the power output, the total work and the oxygen consumption in the second WAnT.

## 2 Materials and methods

The a priori power analysis was performed using G*Power software (version 3.1.9.4) considering a two-way MANOVA test: with an effect size f = 0.87, α = 0.05, and statistical power (1−β) = 0.8, a total of 12 subjects must be enrolled. Estimation of expected effect size is based on Bogdanis et al. (1996), who found a higher mean power output during a second 30″ sprint after an active recovery (603 ± 17 W), compared with passive recovery (589 ± 15 W) [20]. Therefore, twelve well-trained male cyclists (see Table 1) were recruited for this study. The cyclists had a minimum of three years of training experience without interruptions longer than a month and were involved in regular road and mountain bike races. The average weekly training hours was 11.9 ± 2.2, while the mean intensity (Borg 6–20) per training session was 15 ± 2.5 AU [21]. Following a medical health screening, all cyclists provided written informed consent to participate in the study, which was approved by the Ethics Committee of the University of Urbino “Carlo Bo”, Italy, and was conducted in accordance with the Declaration of Helsinki for research with human volunteers. All data were collected during the 2017 competitive season.

**Table 1:** Cyclists' characteristics.

	Age	Height	Weight	BMI	FM	Training per week	HR_max_	VO_2max_	VO_2LT_	70% VO_2LT_
**Mean**	28.08	1.79	70.42	22.03	11.31	11.93	186.24	63.39	51.08	35.76
**SD (**±**)**	6.10	0.07	5.95	1.96	3.50	2.24	2.45	8.14	5.85	4.10

All data are reported as mean and standard deviation. Age: years; Height: m; Weight: kg; BMI: body mass index (kg·m^−2^); FM: fat mass (% of body weight); Training per week: time spent by cyclists training per week (hours); HRmax: maximum heart rate measured during the VO_2_max test (beats per minute, bpm); VO_2max_: maximum VO_2_ assessed during the specific test (ml/kg/min); VO_2LT_: VO_2_ value at lactate threshold (ml/kg/min); 70%VO_2LT_: intensity corresponding at 70% of the VO_2_ at lactate threshold (ml/kg/min).

### 2.1 Experimental design

**Fig. 1:**
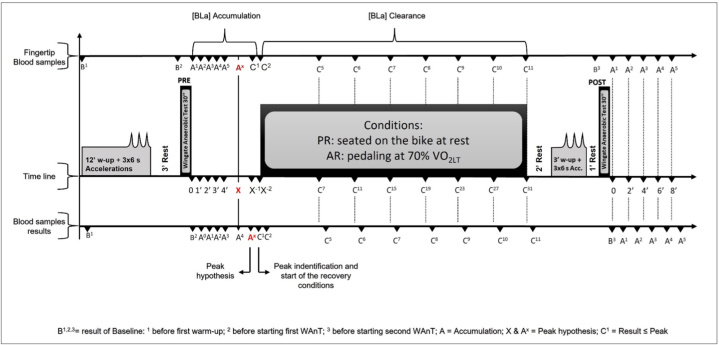
Experimental design.

A crossover study design was used to test the effect of AR vs. PR on mechanical and metabolic responses during a second WAnT. Cyclists were scheduled to undergo three experimental sessions with a one-week interval between sessions to allow them to fully recover. For each session, the cyclists reported to the laboratory well-rested, i.e., without having engaged in strenuous exercise in the previous 48 h and at least 3 h after a light meal. In session 1, the cyclists underwent anthropometric and body composition assessments (via bioimpedance analysis by BIA101 of Akern s. r.l., Italy) as well as maximum oxygen consumption (VO_2_max) and lactate threshold tests as previously described [22]. After a 30-min rest, cyclists underwent an all-out 15-s familiarization trial for the WAnT. In the second and third sessions, cyclists underwent two WAnTs, performing, in random order, 30 min of AR or PR between the WAnTs (the detailed experimental design is represented in Fig. 1).

#### 2.1.1 VO_2_ and blood lactate during the incremental test

The incremental test performed in session 1 started with cyclists pedaling on a cycle ergometer (SRM GmbH, Jülich, Germany) at an intensity equal to 30% of the theoretical VO_2_max estimated by Malek et al. [21], and workload increased every 4 min by 11.67% of VO_2_max until volitional exhaustion [22]. VO_2_ was recorded breath by breath during the entire test using a calibrated Cosmed k4b2 gas analyzer (COSMED, Rome, Italy) and VO_2_max was identified as the maximum value calculated from the 15-breath moving average of VO_2_ over the course of the full test, as described by Robergs et al. [23]. Heart rate (HR) was continuously recorded with the Polar RS-800 heart rate monitor (POLAR, Kempele, Finland) [23]. Blood lactate concentration (BLa) was measured at the end of every 4-min step using the Lactate Pro portable blood lactate analyzer (Arkray, Kioto, Japan) on micro blood samples drawn from the tip of the index finger according to the manufacturer's instructions. The lactate threshold and the corresponding VO_2_LT were determined by using the software developed by Newell et al. [24]. Briefly, this method finds the LT as the intersection of two regression lines fitted to log-transformed data that have the minimum overall residual sum of squares [25].

#### 2.1.2 WAnT testing and recovery protocols

In the second and third sessions, the cyclists underwent a 12-min warm-up [26] at an intensity of 50% of VO_2_ max interspersed with 6-s maximal accelerations at 0 W at the 4th, 7th, and 10th min. The cyclists then rested passively for 3 min before starting the first WAnT. The warm-up and WAnT tests were performed on a mechanically braked Monark Peak Bike (Peak Bike 894, Monark, Vansbro, Sweden) using a standard protocol [27]. Briefly, cyclists pedaled as fast as they could with no brake on the flywheel, and within the first 3 s, a load equal to 10% of their body mass (with friction load equal to 0.098 kp·kg-1) was applied all at once to the flywheel and the cyclists had to maintain maximal pedaling for 30 s. The peak power (PPW, in W), average power (APW, in W), time to reach the peak power (tPP, in ms), and the total work (TWJ, in J) were calculated using the software of the Monark 894 Peak Bike and recorded as performance measures.

#### 2.1.3 VO2 kinetics and blood lactate during WAnTs

To analyze the VO_2_ kinetics during the two WAnTs, the VO_2_ was recorded breath by breath during the entire protocol in both conditions using the same gas analyzer previously described for session 1. Raw VO_2_ data were smoothed using a 15-point-rolling average. For the AR condition, the cyclists pedaled at an intensity that corresponded to 70% of VO_2_LT, as this intensity has been previously shown to maximize lactate clearance [[28], [29], [30]]. For the PR condition instead, the cyclists rested sitting on the bike. Moreover, to verify differences between pre-vs. post-tests and both conditions (AR vs. PR), the highest value of VO_2_ (VO_2_peak) during the WAnTs was also determined. VO_2_ kinetics were modelized using a simple exponential model, with a baseline value [31]. Briefly, the following equation was used: VO_2_ = a + b ∙ e (k∙t); where VO_2_ was the oxygen consumption (dependent variable); t was the independent variable (time, in s); a was the VO_2_ baseline value; b was the amplitude; k was the exponential constant, in particular, k > 0 for the ascending time series (k-A or VO_2_–on kinetics) and k < 0 for the descending phase (k-D or VO_2_–off kinetics). Nonlinear regressions were performed using a Levenberg-Marquardt algorithm [32].

Blood lactate concentration (BLa) was measured every minute following the completion of each WAnT using the same lactate analyzer and procedures as for session 1 (Fig. 1). The resting BLa was measured before the first warm-up, whilst the baseline BLa (BLa-baseline) was measured just before every WAnT. To accurately identify the beginning of the lactate clearance phase, BLa samples were collected every minute following the completion of the first WAnT until a BLa value equal or lower than the previous one was reached. The highest value was considered as the peak BLa (BLa-peak). The identification of the BLa-peak was out of phase by 2 min with its actual attainment because of the time needed by the lactate analyzer to give a result [22] (Fig. 1). After reaching the first BLa-peak, the cyclists began the AR at 70% of the VO_2_LT or the PR seated on the bike for 31 min. In both conditions, the blood lactate clearance was measured at the 7th, 11th, 15th, 19th, 23rd, 27th, and 31st min (Fig. 1). Subsequently, the cyclists rested for 2 min and then performed a 3-min warm-up with 6-s maximal accelerations with 0 W at the end of each minute. Cyclists then rested for 1 min before starting the second WAnT [33,34]. The BLa-peak of the second WAnT was determined by collecting blood samples immediately after the end of the test and every 2 min until the 8th minute.

### 2.2 Statistical analysis

Descriptive analyses were reported as means ± SD. A 2-way Multivariate analysis of variance (MANOVA) was used to test the effect of the recovery condition (active vs. passive) and moments (pre vs. post). The dependent variables were the: VO_2_peak, BLa-baseline, BLa-peak, PPW, tPP, APW, and TWJ. Also, the k parameter, derived from nonlinear regression using an exponential equation, was analyzed with MANOVA test. In addition, the effect size (ES- eta squared value) was reported; eta-squared ranges from 0 to 1 and indicates the proportion of overlap between the predictor and outcome variable. In other words, 0 indicates no association between the variables, and values close to 1 indicate a high degree of association. BLa values were compared at each time point using ANOVA for repeated measures to verify differences in lactate clearance in both recovery conditions (AR vs. PR). A post-hoc based *t*-test for paired data with the Bonferroni correction was used to detect significant differences between pairs. Statistical significance was set at 5%. The experimental design is shown in Fig. 1.

## 3 Results

**Fig. 2:**
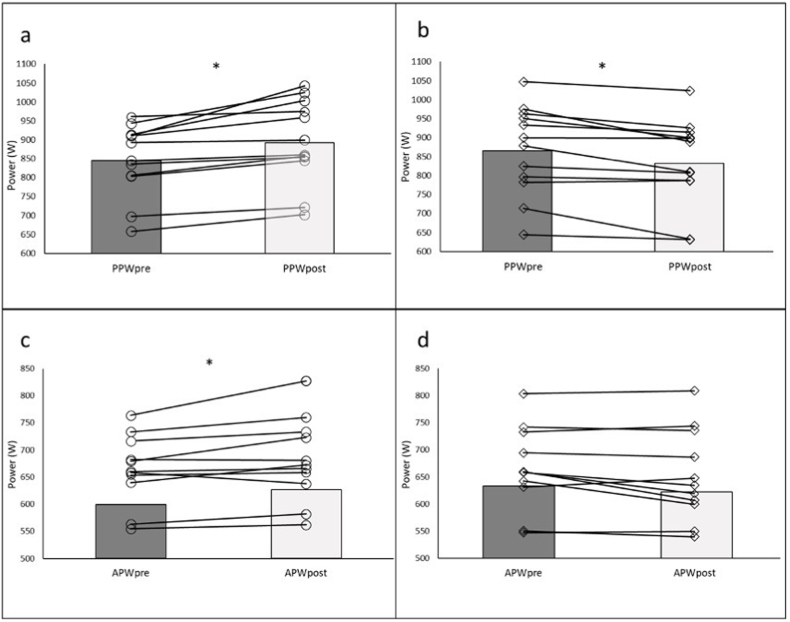
A: PPW pre vs. PPW post – AR; b: PPW pre vs. PPW post – PR; c: APW pre vs APW post – AR; d: APW pre vs APW post – PR; *: significant differences (a and c: p < 0.001; b: p < 0.05).

The analyses of the effects of the active and passive condition and recovery values (pre-post) showed no differences in the VO_2_peak, BLa-peak or tPP. However, we found significant differences in the BLa-baseline post, PPW, APW, and TWJ. The mean values, standard deviation, p values and effect sizes are reported in Table 2. The differences in PPW and APW between pre and post in both AR and PR conditions are shown in Fig. 2. In particular, the PPW and APW were found to be significantly higher post AR compared to post PR. In particular, the PPW was 5.6% higher than pre in the AR con-dition, while in the same condition, the APW was 2.6% higher than pre. For the PR, the PPW was significantly lower (−3.9%) in post vs pre, while no significant difference was found for the APW (−1.6%). Finally, the total work carried out during the WAnT, ex-pressed as TWJ, was significantly higher after the AR phase than it was after the PR.

**Table 2:** Analysis of all pre and post WAnT variables in active and passive recovery conditions.

	AR pre	PR pre	p AR vs. PR pre (ES)	AR post	PR post	p AR vs. PR post (ES)	p interaction (ES)
**VO**_**2peak**_**(ml/kg/min)**	54.72 ± 5.47	52.5 ± 4.40	0.083 (0.32)	54.03 ± 5.60	51.1 ± 6.70	0.182 (0.21)	0.668 (0.02)
**BLa-baseline (mmol/L)**	1.69 ± 0.56	1.69 ± 0.48	1.000 (0.00)	2.06 ± 0.92	4.45 ± 1.18	<**0.001 (0.75)**	<**0.001 (0.77)**
**BLa-peak (mmol/L)**	12.96 ± 2.4	13.36 ± 2.35	0.068 (0.42)	13.26 ± 2.05	13.16 ± 2.10	0.815 (0.01)	0.077 (0.33)
**PPW (W)**	848.0 ± 94.9	867.3 ± 118.1	0.124 (0.26)	895.3 ± 110.8	833.5 ± 116.7	<**0.001 (0.79)**	<**0.001 (0.85)**
**tPP (ms)**	3229.6 ± 1518.0	3359.3 ± 1838.0	0.700 (0.02)	3897.1 ± 2300.2	3491.7 ± 1917.2	0.100 (0.24)	0.200 (0.22)
**APW (W)**	664.1 ± 63.9	665.5 ± 77.0	0.811 (0.01)	682.0 ± 76.6	652.12 ± 83.9	<**0.001 (0.79)**	<**0.001 (0.84)**
**TWJ (J)**	17,172 ± 4852	17,246 ± 4854	0.650 (0.03)	17,568 ± 4927	17,110 ± 4631	0.140 (0.24)	**0.046 (0.39)**

All data are reported as mean and standard deviation; VO2peak: is the highest value (ml/kg/min) of VO2 during the Wingate anaerobic tests (WAnT); BLa-baseline: is the blood lactate concentration before each WAnT (in mmol/L); BLa-peak: is the highest value of blood lactate concentration after each WAnT (in mmol/L); PPW: is the highest value of power measured during each WAnT (in W); tPP: is the time to reach the highest value of power during the WanTs (in ms); APW: is the average power during the 30 s of each WAnT (in W); TWJ: is the energy of the total work produced throughout the tests (in J). AR pre: are the values assessed before the active recovery condition; PR pre: are the values assessed before the passive recovery condition; AR post: are the values measured after the active recovery condition; PR post: are the values measured after the passive recovery condition; p AR vs. PR pre (ES): represents the significance (pearson and effect size) be-tween PR and AR before the recovery condition; p AR vs. PR post (ES): represents the significance (pearson and effect size) between PR and AR after the recovery condition; p interaction (ES): is the value of significance (pearson) and Effect size (ES) between the Ar and PR conditions.

**Fig. 3:**
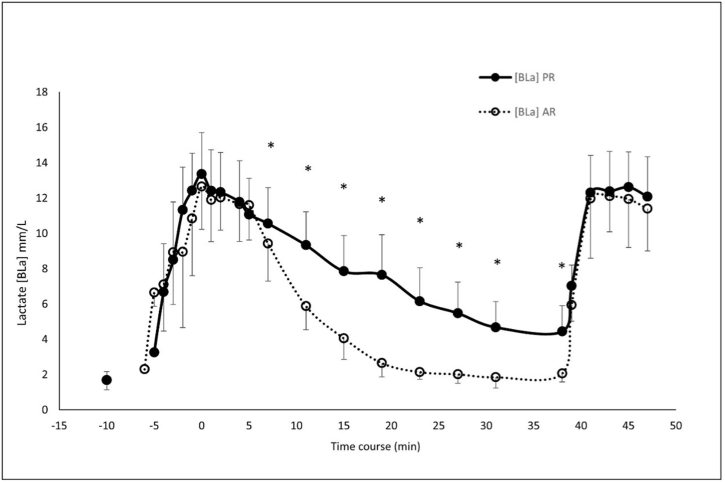
Blood lactate samples during both moderate intensity active (AR) and passive (PR) experimental recovery conditions. Time 0 corresponds to the peak lactate achievement after the first WAnT. *: significant differences at 7th, 11th, 15th, 19th, 23rd, 27th, and 31st minutes and immediately before the start of WAnT post at the 38th minute (p < 0.05).

**Fig. 4:**
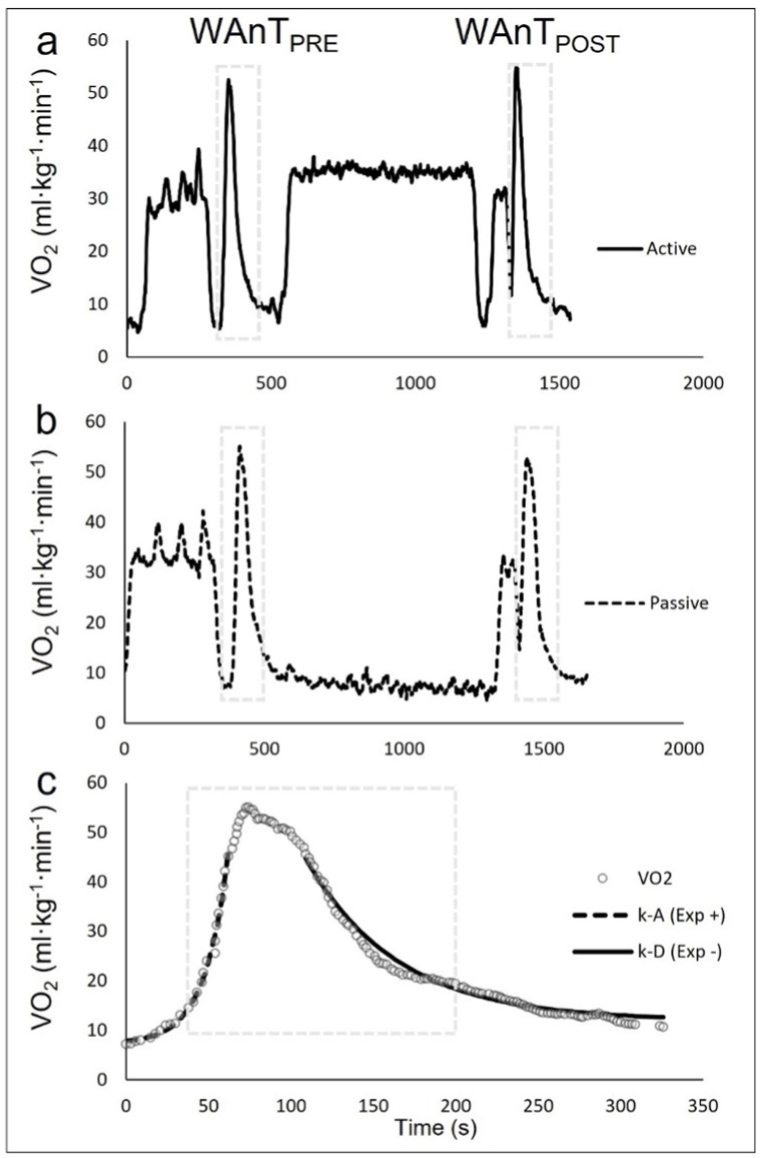
VO_2_ values during the experimental protocol for a representative cyclist; a: AR condition (70%VO_2_LT). b: PR condition (seated on the bike); WAnT PRE and WAnT POST represent the first and second WAnT; c: Representation of the kinetic analysis of VO_2_ during a WAnT: k-A is the positive (ascending part) and k-D is the negative (descending part) of the exponential model fitting function.

Regarding the analysis of BLa, the BLa-baseline was lower in AR vs. PR post with a large effect size of the interaction of the conditions, while no difference was found at pre. No differences were found in the BLa-peak in both conditions at pre and post. Moreover, we found no differences in the pre vs. post or between the two conditions. On the other hand, as expected, there was a significant difference between the PR and AR conditions for lactate clearance. Specifically, in the AR, lactate levels were already significantly lower at the 7th minute from the peak and remained so until the end of the active recovery at the 31st min. Furthermore, lactate levels remained significantly higher in the PR condition than in the AR until the 38th minute when the baseline blood sample was collected before the second WAnT (see Fig. 3). The positive (ascending part or k-A) and negative (descending part or k-D) exponential function model fitting with the curve of the VO_2_ during WAnTs showed a R2 >0.96 and CVs of 38.9% and 33.3%, respectively. The analysis of the VO_2_ kinetics during WAnTs pre vs. post showed no significant differences both in PR and AR conditions. In particular, we report the Δ of post minus pre for both k-A and k-D and the p-value of the pre vs. post in every condition: k-A PR Δ post-pre 0.007 ± 0.0, p > 0.05; k-A AR Δ post-pre 0.005 ± 0.00, p > 0.05; k-D PR Δ post-pre 0.000 ± 0.0, p > 0.05; k-D AR Δ post-pre 0.001 ± 0.00, p > 0.05. Moreover, no significant interaction was found between conditions (p > 0.05) (the representation of VO_2_ kinetics is shown in Fig. 4 c).

## 4 Discussion

The main objective of this study was to compare the effects of a moderate active recovery vs. a passive recovery on the mechanical and metabolic variables during the execution of two WAnTs in cyclists. Our results show that an AR strategy conducted at 70% of VO_2_LT improved lactate clearance prior to the second WAnT (Fig. 3) and the mechanical variables during the second WAnT (Fig. 2). However, and contrary to our hypothesis, VO_2_peak and VO_2_ kinetics remained unaffected by the recovery method during the second WAnT.

### 4.1 VO_2_peak and VO_2_ kinetics

Previous studies have shown that VO_2_ kinetics can be affected by the intensity at which prior activity was performed [6,8]. However, these studies aimed to test the effectiveness of different warm-up intensities that preceded a maximal effort, and therefore elicited different physiological conditions in terms of VO_2_ kinetics immediately before the maximal test. In contrast, in our study, the aim was to analyze the effects of active or passive recovery between two short-duration all-out anaerobic activities. Therefore, our protocol was methodologically designed to ensure that in the two recovery conditions the cyclists had similar baseline oxygen consumption before each maximal test (Fig. 4a and b). Contrary to our results, Fujita et al. [17] found a significantly higher VO_2_ peak in the second sprint after the AR condition only. However, their protocol was different because it did not include any warm-up before the maximal exercises. We consequently speculate that the AR condition in their study also served as a warm-up for the second maximal exercise, and therefore a higher performance and VO_2_peak would be expected. Bogdanis et al. found an increase on average VO_2_ from the first to a second 30-s sprint after a 4-min AR [20]. This result, however, can be explained by the very short duration of the recovery phase in their protocol (only 4 min) and by the different conditions before the 1st and 2nd sprints. Indeed, all their subjects rested passively for 5 min between the end of the warm-up and the beginning of the first sprint, whilst the subjects who recovered actively pedaled continuously until the very start of the second sprint. This implied different starting VO_2_ levels between sprints that not only had a substantial impact on the higher average VO_2_ measured in the second sprint but also between the active and passive conditions. In our protocol, to ensure an optimal warm-up and minimize the time between the recovery phase and the start of the second WAnT, we designed a warm-up phase both for the pre and post WAnTs with different time durations (12 min before the first WAnT vs. 3 min before the second) and resting times (3 min before the first WAnT vs. 1 min before the second). However, these differences were sufficient to not significantly alter neither the ascending and descending phases of VO_2_ kinetics nor VO_2_ peak between the first and second WAnTs. As a result, we hypothesize that the main factors underlying the VO_2_ peak and kinetics results might be attributable to the long recovery period between WAnTs (about 40 min, including active or passive recovery, second warm-up, and resting periods) and by the proper warm-up involved before each WAnT to ensure the same starting conditions both after passive and active recoveries. However, more research is needed to confirm this hypothesis.

### 4.2 Lactate clearance

As expected, the lactate clearance in the AR performed at 70% of the LT was significantly greater than in the PR as early as from the seventh minute after the BLa-peak. In contrast, other researchers that used lower recovery intensities did not find the same results after all-out sprints. Koizumi et al. found a difference in BLa between AR and PR only after 20 min of pedaling at 30% of the ventilatory threshold [16]. Furthermore, Fujita et al. did not find any differences between PR and AR at 50% of the ventilatory threshold [17]. The lower intensity of the AR in the former study and the longer duration of the maximal effort in the latter (40 s vs. 30 s) might explain these discrepancies. Indeed, it has been shown that intensity between 80% and 100% of the lactate threshold optimizes lactate recovery rate both in cycling [18] and in running [30], which is only slightly higher than the intensity used in our study. Therefore, the AR used in the current study allowed to perform the post WAnT with a lower basal blood lactate concentration than in the PR, improving recovery capability from the first WAnT. Considering that the BLa-peak during the second WAnTs were not different for both AR and PR conditions, we may hypothesize that the glycolytic contribution was enhanced during the AR [31], triggering a dual effect of both recovery and potentiation.

### 4.3 Total work, average and peak power

Along with the improved lactate clearance, we also found an increase in mechanical variables (PPW, APW and TWJ) after the AR condition only, and a decrease in PPW following the PR. In particular, the enhanced glycolytic activity in the second WAnT after the AR likely contributed to the higher APW and TWJ by allowing a higher power production after the first 5 s [31,35]. In this regard, Koizumi et al. (2011) [16] previously suggested that during the latter phase of a WAnT preceded by continuous aerobic exercise, there is an increase in oxidative enzyme activity, and this could also contribute to the increased APW and TWJ found in our study. Regarding the increase in PPW found after the AR, similar results were found by Koizumi et al. (2011) [16] according to which it can be speculated that a higher muscle oxygenation level during AR could be responsible for the improved recovery of the fast twitch fibers, thus contributing to the higher PPW during the second WAnT. However, despite these hypotheses seeming to explain an increase in total work and in average power in the WAnT preceded by AR, several doubts remain about the cause of the increased PPW. Indeed, although the better recovery of the fast twitch fibers thanks to the AR could explain the differences in PPW in the second WAnT between both conditions, it cannot explain the improved PPW found between pre and post WAnTs. As expected, in our study the PPW during the WAnTs was reached within the first 5 s (see Table 2). As shown by several authors [31,35], the energy contribution of this phase (0–5 s) is predominantly alactic although the glycolytic energy system has already started. Indeed, the maximal glycolysis rate is reached between the 5th and 10th seconds and then remains as the main energy source until the end of the WAnT [29]. In contrast, the aerobic energy contribution starts from the 7th second and contributes between 16 and 18.6% of total energy production only [31,35]. For these reasons, both the aerobic and anaerobic lactic energy contributions can't explain the increase in PPW, and therefore other mechanisms must be involved. In another study, 6 min of warm-up at almost VO_2_max intensity (the so-called priming exercise) induced an increase in PPW after 30 min, and the authors suggested that the potential mechanisms could be changes in hormones (epinephrine and adrenaline) and the post-activation potentiation (PAP) [36]. In particular, the PAP mechanism could be involved also in our AR strategy by increasing calcium sensitivity and myosin light chain phosphorylation, responsible for the increase in the rate of cross-bridging [37,38]. Moreover, other authors have also suggested that a previous activity that induces a selective glycogen depletion from slow fibers might improve the recruitment of fast twitch fibers in a subsequent high-intensity activity [15,39].

To the best of our knowledge, this is the first study to analyze the effects of active recovery on all-out sprint exercises in cyclists rather than in active healthy subjects. Although a WAnT does not exactly match the sprint requirements of a cycling race and the intensity and duration of the recovery can hardly be selected during an actual race, our results might help cyclists and coaches to develop adequate racing strategies. For instance, a cyclist could use a first attack not to get in a breakaway or to win an intermediate sprint but to activate the physiological mechanisms that will boost the power production at the finish line. Something similar happened in the second stage of the 2021 Tour de France, when cyclist Mathieu van der Poel attacked on a small climb at 16.9 km from the finish line to get a time bonus. He then used the following downhill to recover and about 20 min later he won the stage (and also the leader's jersey) on the same climb with a final attack at 800 m from the finish line.

## 5 Conclusions

This study is the first to investigate the effects of an AR strategy between two all-out anaerobic exercises in trained cyclists. Our results show that 30 min of active recovery at 70% of lactate threshold increased the performance indices in a subsequent WAnT in terms of external load without changing the VO_2_peak, VO_2_ kinetics nor BLa-peak in trained cyclists. This study might help to improve race strategies and training in competitive cyclists. Future research should focus on optimal recovery intensities and durations, as well as the number of sprints that can benefit from the recovery and repeated warm-up strategies in different sports settings.

## Funding

This research received no external funding

## Institutional review board statement

The study was conducted in accordance with the Declaration of Helsinki and approved by the Institutional Ethics Committee of University of Urbino (CESU-31-2017).” for studies involving humans.

## Informed consent statement

Informed consent was obtained from all participants involved in the study.

## Author contribution statement

Marco Gervasi: Eneko Fernández-Peña: Conceived and designed the experiments; Analyzed and interpreted the data; Contributed reagents, materials, analysis tools or data; Wrote the paper.

Antonino Patti: Analyzed and interpreted the data; Contributed reagents, materials, analysis tools or data; Wrote the paper.

Piero Benelli: Davide Sisti: Performed the experiments.

Johnny Padulo: Daniel Boullosa: Contributed reagents, materials, analysis tools or data.

## Data availability statement

Data will be made available on request.

## Additional information

No additional information is available for this paper.

## Declaration of competing interest

The authors declare that they have no known competing financial interests or personal relationships that could have appeared to influence the work reported in this paper.

## References

[1] Gray S.R., Soderlund K., Watson M., Ferguson R.A. (2011). Skeletal muscle ATP turnover and single fibre ATP and PCr content during intense exercise at different muscle temperatures in humans. Pflügers Archiv.

[2] Pearce A.J., Rowe G.S., Whyte D.G. (2012). Neural conduction and excitability following a simple warm up. J. Sci. Med. Sport.

[3] Poole D.C., Jones A.M. (2012). Oxygen uptake kinetics. Compr. Physiol..

[4] Sale D.G. (2002). Postactivation potentiation: role in human performance. Exerc. Sport Sci. Rev..

[5] McGowan C.J., Pyne D.B., Thompson K.G., Rattray B. (2015). Warm-up strategies for sport and exercise: mechanisms and applications. Sports Med..

[6] Burnley M., Doust J.H., Carter H., Jones A.M. (2001). Effects of prior exercise and recovery duration on oxygen uptake kinetics during heavy exercise in humans. Exp. Physiol..

[7] Burnley M., Jones A.M., Carter H., Doust J.H. (1985). Effects of prior heavy exercise on phase II pulmonary oxygen uptake kinetics during heavy exercise. J. Appl. Physiol..

[8] Gerbino A., Ward S.A., Whipp B.J. (1985). Effects of prior exercise on pulmonary gas-exchange kinetics during high-intensity exercise in humans. J. Appl. Physiol..

[9] Buchheit M., Cormie P., Abbiss C.R., Ahmaidi S., Nosaka K.K., Laursen P.B. (2009). Muscle deoxygenation during repeated sprint running: effect of active vs. passive recovery. Int. J. Sports Med..

[10] Toubekis A.G., Adam G.V., Douda H.T., Antoniou P.D., Douroundos I.I., Tokmakidis S.P. (2011). Repeated sprint swimming performance after low- or high-intensity active and passive recoveries. J. Strength Condit Res..

[11] Dupont G., Blondel N., Berthoin S. (2003). Performance for short intermittent runs: active recovery vs. passive recovery. Eur. J. Appl. Physiol..

[12] Connolly D.A., Brennan K.M., Lauzon C.D. (2003). Effects of active versus passive recovery on power output during repeated bouts of short term, high intensity exercise. J. Sports Sci. Med..

[13] Spierer D.K., Goldsmith R., Baran D.A., Hryniewicz K., Katz S.D. (2004). Effects of active vs. passive recovery on work performed during serial supramaximal exercise tests. Int. J. Sports Med..

[14] Nalbandian H.M., Radak Z., Takeda M. (2017). Active recovery between interval bouts reduces blood lactate while improving subsequent exercise performance in trained men. Sports.

[15] Gervasi M., Calavalle A.R., Amatori S., Grassi E., Benelli P., Sestili P., Sisti D. (2018). Post-activation potentiation increases recruitment of fast twitch fibers: a potential practical application in runners. J. Hum. Kinet..

[16] Koizumi K., Fujita Y., Muramatsu S., Manabe M., Ito M., Nomura J. (2011). Active recovery effects on local oxygenation level during intensive cycling bouts. J. Sports Sci..

[17] Fujita Y., Koizumi K., Sukeno S., Manabe M., Nomura J. (2009). Active recovery effects by previously inactive muscles on 40-s exhaustive cycling. J. Sports Sci..

[18] Iwahara F., Ito M., Asami T. (2003). Effect of cooling down on blood lactate removal and anaerobic workout in exhaustive cycle ergometer exercise. Jpn. J. Phys. Fit. Sports Med..

[19] Ardigo L.P., Palermi S., Padulo J., Dhahbi W., Russo L., Linetti S., Cular D., Tomljanovic M. (2020). External responsiveness of the SuperOp(TM) device to assess recovery after exercise: a pilot study. Front Sports Act Living.

[20] Bogdanis G.C., Nevill M.E., Lakomy H.K., Graham C.M., Louis G. (1996). Effects of active recovery on power output during repeated maximal sprint cycling. Eur. J. Appl. Physiol. Occup. Physiol..

[21] Malek M.H., Housh T.J., Berger D.E., Coburn J.W., Beck T.W. (2005). A new non-exercise-based Vo2max prediction equation for aerobically trained men. J. Strength Condit Res..

[22] Lucertini F., Gervasi M., D'Amen G., Sisti D., Rocchi M.B.L., Stocchi V., Benelli P. (2017). Effect of water-based recovery on blood lactate removal after high-intensity exercise. PLoS One.

[23] Robergs R.A., Dwyer D., Astorino T. (2010). Recommendations for improved data processing from expired gas analysis indirect calorimetry. Sports Med..

[24] Newell J., Higgins D., Madden N., Cruickshank J., Einbeck J., McMillan K., McDonald R. (2007). Software for calculating blood lactate endurance markers. J. Sports Sci..

[25] Lundberg M.A., Hughson R.L., Weisiger K.H., Jones R.H., Swanson G.D. (1986). Computerized estimation of lactate threshold. Comput. Biomed. Res..

[26] Racinais S., Blonc S., Hue O. (2005). Effects of active warm-up and diurnal increase in temperature on muscular power. Med. Sci. Sports Exerc..

[27] Bar-Or O. (1987). The Wingate anaerobic test. An update on methodology, reliability and validity. Sports Med..

[28] Meyer T., Gabriel H.H., Kindermann W. (1999). Is determination of exercise intensities as percentages of VO2max or HRmax adequate?. Med. Sci. Sports Exerc..

[29] Devlin J., Paton B., Poole L., Sun W., Ferguson C., Wilson J., Kemi O.J. (2014). Blood lactate clearance after maximal exercise depends on active recovery intensity. J. Sports Med. Phys. Fit..

[30] Menzies P., Menzies C., McIntyre L., Paterson P., Wilson J., Kemi O.J. (2010). Blood lactate clearance during active recovery after an intense running bout depends on the intensity of the active recovery. J. Sports Sci..

[31] Beneke R., Pollmann C., Bleif I., Leithauser R.M., Hutler M. (2002). How anaerobic is the wingate anaerobic test for humans?. Eur. J. Appl. Physiol..

[32] Kanzow C., Yamashita N., Fukushima M. (2005). WITHDRAWN: Levenberg–Marquardt methods with strong local convergence properties for solving nonlinear equations with convex constraints. J. Comput. Appl. Math..

[33] Bernard T., Giacomoni M., Gavarry O., Seymat M., Falgairette G. (1998). Time-of-day effects in maximal anaerobic leg exercise. Eur. J. Appl. Physiol. Occup. Physiol..

[34] Racinais S., Hue O., Hertogh C., Damiani M., Blonc S. (2004). Time-of-day effects in maximal anaerobic leg exercise in tropical environment: a first approach. Int. J. Sports Med..

[35] Smith J.C., Hill D.W. (1991). Contribution of energy systems during a Wingate power test. Br. J. Sports Med..

[36] Ktenidis C.K., Margaritelis N.V., Cherouveim E.D., Stergiopoulos D.C., Malliou V.J., Geladas N.D., Nikolaidis M.G., Paschalis V. (2021). Priming exercise increases Wingate cycling peak power output. Eur. J. Sport Sci..

[37] Boullosa D., Del Rosso S., Behm D.G., Foster C. (2018). Post-activation potentiation (PAP) in endurance sports: a review. Eur. J. Sport Sci..

[38] Grange R.W., Vandenboom R., Xeni J., Houston M.E. (1985). Potentiation of in vitro concentric work in mouse fast muscle. J. Appl. Physiol..

[39] Krustrup P., Soderlund K., Mohr M., Bangsbo J. (2004). Slow-twitch fiber glycogen depletion elevates moderate-exercise fast-twitch fiber activity and O2 uptake. Med. Sci. Sports Exerc..

